# Novel Monthly Quality Assurance Regimen and 5-Year Analysis Using a Proton Metrology System

**DOI:** 10.14338/IJPT-23-00004.1

**Published:** 2023-10-30

**Authors:** Ozgur Ates, Jackie Faught, Fakhriddin Pirlepesov, David Sobczak, Chia-ho Hua, Thomas E. Merchant

**Affiliations:** Department of Radiation Oncology, St. Jude Children’s Research Hospital, Memphis, TN, USA

**Keywords:** XRV-100, XRV-124, proton metrology, proton monthly QA

## Abstract

**Purpose:**

To develop a novel, monthly quality assurance (QA) regimen for a proton therapy system that uses 2 custom phantoms, each housing a commercial scintillator detector and a charge-coupled device camera. The novel metrology system assessed QA trends at a pediatric proton therapy center from 2018 to 2022.

**Materials and Methods:**

The measurement system was designed to accommodate horizontal and vertical positioning of the commercial device and to enable gantry and couch isocentricity measurements (using a star shot procedure), proton spot profile verification, and imaging and radiation congruence tests to be performed simultaneously in the dual-phantom setup. Gantry angles and proton beam energies were varied and alternated each month, using gantry angles of 0°, 30°, 60°, 90°, 120°, 150°, and 180° and discrete beam energies of 69.4, 84.5, 100, 139.1, 180.4, 200.4, and 221.3 MeV after radiographic verification. A total of 1176 individual monthly QA measurements of gantry and couch isocentricity, spot size, and congruence were analyzed.

**Results:**

Gantry and couch star shot measurements showed beam isocentricities of 0.3 ± 0.2 mm and 0.2 ± 0.2 mm, respectively, which were within the threshold of 1.0 mm. Spot sizes for each discrete energy were within the threshold of ± 10% of the baseline values for all 3 proton rooms. The imaging and radiation coincidence test results for the 1176 individual monthly QA measurements were 0.5 mm for the 50^th^ percentile and 1.2 mm (the clinical threshold) for the 97.6^th^ percentile.

**Conclusions:**

Integrating a commercial device with custom phantoms improved the quality of proton system checks compared with previous methods using radiochromic films, loose ball bearings, and foam. The scheme of alternating beam angles with discrete energies in the monthly QA-enabled, clinically meaningful verification of beam energy and gantry angle combinations while the machine performance and accuracy were being checked.

## Introduction

In proton therapy, pristine Bragg peaks are typically generated within a range of 70 to 250 MeV, with a narrow beam size of 5 to 15 mm [1]. The proton energies are varied to form the spread-out Bragg peak at the desired depth. The number of discrete beams with various ranges and sizes adds to the complexity of proton beam delivery compared with linac-based treatment using photon beams in the range of 6 to 18 MV. Therefore, a dedicated quality assurance (QA) program is required to verify the proton beam monitor units (MU) (ie, the dose), position, and size with respect to the machine specifications accepted at the time of commissioning.

In 2019, the American Association of Physicists in Medicine Task Group (TG) 224 published a report on QA for proton therapy machines, which recommended various procedures for assessing proton beam delivery mechanisms, beam parameters, and instrumentation [2]. The principles discussed in the TG-224 report were similar to those for linac-based QA outlined in the previously published TG-40 [3], TG-142 [4], and TG-179 [5] reports. However, the precise delivery of proton therapy, which relies on many discrete proton beam energies, requires sophisticated radiation instrumentation and comprehensive verification of the beam parameters in a periodic QA workflow [6].

Several proton therapy centers have reported using phantoms constructed in-house for routine machine QA procedures [7–10]. These in-house phantoms were retrofitted to existing commercial devices and used to measure proton spot properties in terms of MU, size, position, and range. A commercial scintillator detector cone with a charge-couple device camera, namely the Logos Systems XRV-100 or XRV-124 (Logos Systems International, Scotts Valley, CA), can both detect spot profiles and is well-visualized with radiographic imaging. Two groups have reported using XRV detectors in the end-to-end testing of pencil-beam scanning proton therapy machines for spot profiles and for verifying the imaging and radiation coincidence [11, 12].

According to Logos Systems International (personal communication), more than 150 XRV-100/124 units have been shipped worldwide, mostly to proton therapy centers. To our knowledge, we are the first proton center to use an XRV scintillator detector in a vertical position with the aid of an in-house phantom to enable measurements of couch isocentricity and the only center to have designed a monthly QA regimen based on alternating the varying proton beam energies and gantry angles for each monthly QA cycle.

## Materials and Methods

**Figure 1.**
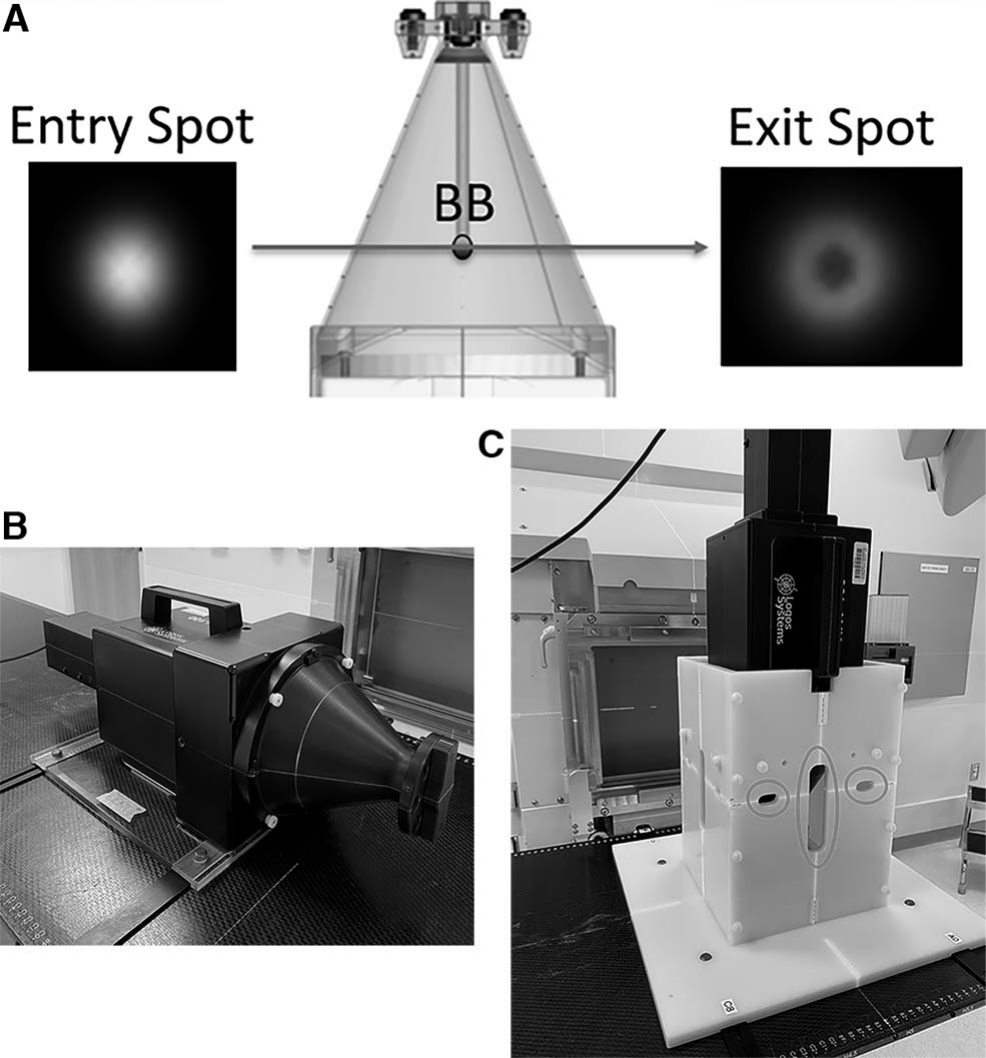
(A) The proton entry and exit spots in the XRV detector. (B and C) The phantom setups with the XRV device in the horizontal (B) and vertical (C) positions for gantry and couch isocentricity measurements, respectively. The slits of the vertical phantom were shown within red circles on 1 face of the phantom. These slits enable the passage of proton beams. BB: Ball bearing.

A novel monthly QA regimen for proton therapy was established based on 2 custom phantoms, which held a commercial XRV-100 conical scintillator detector. The XRV detector that weighs 8 kg has scintillator phosphor layers on the interior surface of the cone. When the scintillator is excited by ionizing particles, such as protons, the charge-coupled device camera captures the beam position and the brightness of the particle track, calculates the width of the beam within the cone, and identifies the entry and exit spots as shown in **Figure 1A**. A tungsten ball bearing (BB) located in the tip of a probe along with the central axis of the cone enables alignment via radiographic imaging for end-to-end testing of the radiation and imaging isocenter coincidence with submillimetric accuracy.

The XRV proton metrology system at our center was augmented to accommodate horizontal and vertical positioning of the XRV device to enable gantry and couch isocentricity measurements (by the star shot procedure), proton spot profile verification, and imaging and radiation congruence tests to be performed simultaneously in a dual-phantom setup as shown in **Figures 1B** and **1C**. Spot size, gantry isocentricity, and congruence were all measured simultaneously in a single, horizontal phantom setup. The horizontal phantom weighed 0.75 kg and was made of polycarbonate material. The horizontal phantom allowed the user to precisely align the XRV device with the designated notch positions on the CIVCO couch top (CIVCO Radiotherapy LLC, Orange City, Iowa, USA). This horizontal setup was achieved using 2 index bars, ensuring a systematic alignment and increased accuracy.

Gantry angles and proton beam energies were varied and alternated each month, using gantry angles of 0°, 30°, 60°, 90°, 120°, 150°, and 180° and discrete beam energies of 69.4, 84.5, 100, 139.1, 180.4, 200.4, and 221.3 MeV after radiographic positional verification with 2D X-rays. For example, the beam energy of 69.4 MeV was delivered at a gantry angle 0° in the first month and 30° in the next month in an alternating fashion.

For couch isocentricity measurements, the homemade vertical phantom enabled the commercial XRV device to be used in the vertical position, which was not possible with the device as originally designed. The vertical phantom weighed 4.7 kg and was made of polyethylene material. The vertical phantom contained open slits at 30° increments for star shot measurements at couch angles of 180°, 210°, 240°, 270°, 300°, 330°, and 360° while the gantry was at a 90° angle. The open slits allow proton beams to pass through, while the vertical phantom, fixed to the table, rotates every 30° increment. The vertical phantom is suitable for all gantry rooms, as the beam remains fixed while the couch rotates isocentrically. Without this vertical phantom, it would not be feasible to administer beams that are perpendicular to the central axis of the XRV-100 cone for couch isocentricity measurements. A single energy of 100 MeV was used to perform couch isocentricity or star shot measurements while rotating the couch from 180° to 360°, totaling 7 beam irradiations for each 30° increment to cover the full excursion of couch rotations.

A total of 1176 individual monthly QA measurements of gantry and couch isocentricity, spot size, and radiation and imaging congruence were analyzed. The measurements were collected from 2018 to 2022 in 2 half-gantry rooms (G1 and G2) and a fixed gantry room, using mini and standard beams from a Hitachi PROBEAT-V pencil-beam scanning proton therapy system (Hitachi Ltd., Tokyo, Japan). Hitachi’s robotic 6–degrees of freedom couch was used in the measurements with the capability of 180° rotation.

A mini beam was created by integrating a vertical scraper, leading to smaller spot sizes than the standard beam used in the G1 and G2 rooms. The mini beam, exclusively employed in the fixed gantry room, was extracted from the synchrotron and directed through a thinner vacuum window positioned within the scanning nozzle. The spot size refers to the total width of the beam at half of its maximum intensity, commonly known as the full width at half maximum. The full width at half maximum has been adopted as the most accurate measurement to represent the diameter of the beam and describe the spot size.

## Results

**Figure 2.**
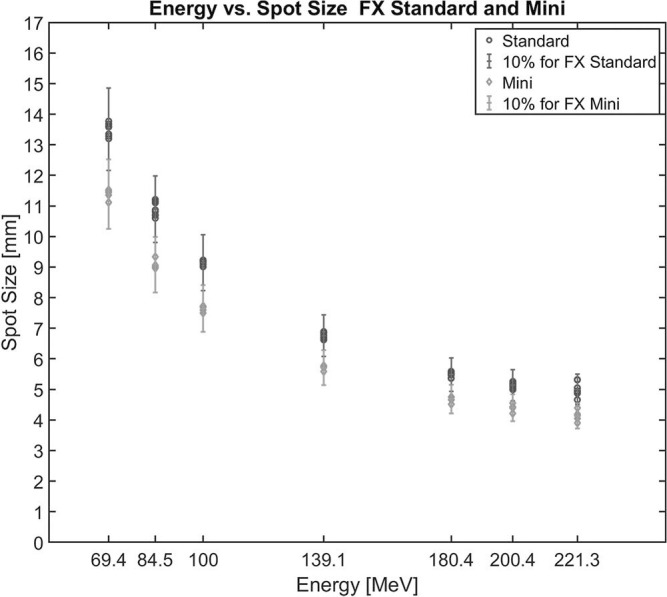
Spot size measurements using standard and mini beams in the FX room for all 7 beam energies used for monthly QA, with error bars showing ± 10% deviation from the values at commissioning. FX: fixed-gantry room.

Spot size measurements for 7 distinct energies delivered at a 90° angle in the fixed gantry room using standard and mini beams showed less than 10% deviation compared with the values at commissioning, which is within the clinical threshold (**Figure 2**).

**Figure 3.**
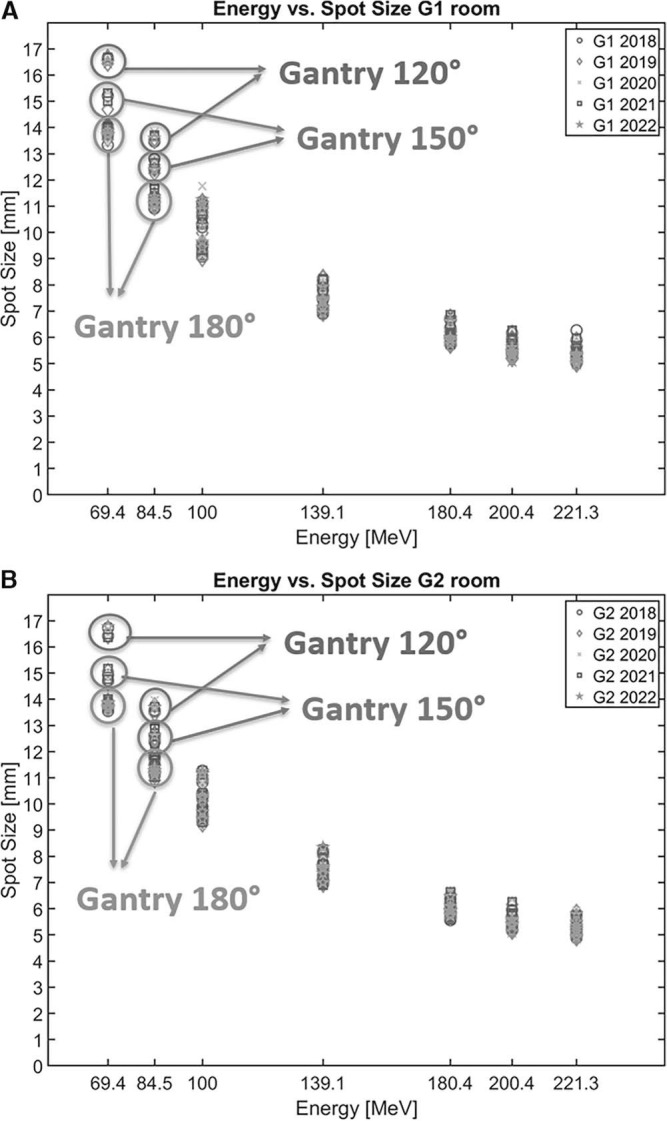
Spot sizes for the seven beam energies as measured in the G1 (A) and G2 (B) rooms, color-coded by year from 2018 to 2022.

Spot size measurements for the 2 half-gantry rooms (G1 and G2) were also within 10% of the baseline values determined at commissioning. However, the combination of the varying gantry angles with the alternating beam energies resulted in posterior (180°) or posterior-oblique (120° and 150°) beams that intersected the couch, experiencing a range shift in the beam energy or momentum, causing larger spot sizes (**Figure 3**). The spread of the spot sizes was more pronounced or coarser with the lower-energy beams. In contrast, the higher-energy beams resulted in finer spot sizes because there was less multiple scattering, which is an expected physical effect observed in G1 and G2 rooms.

**Figure 4.**
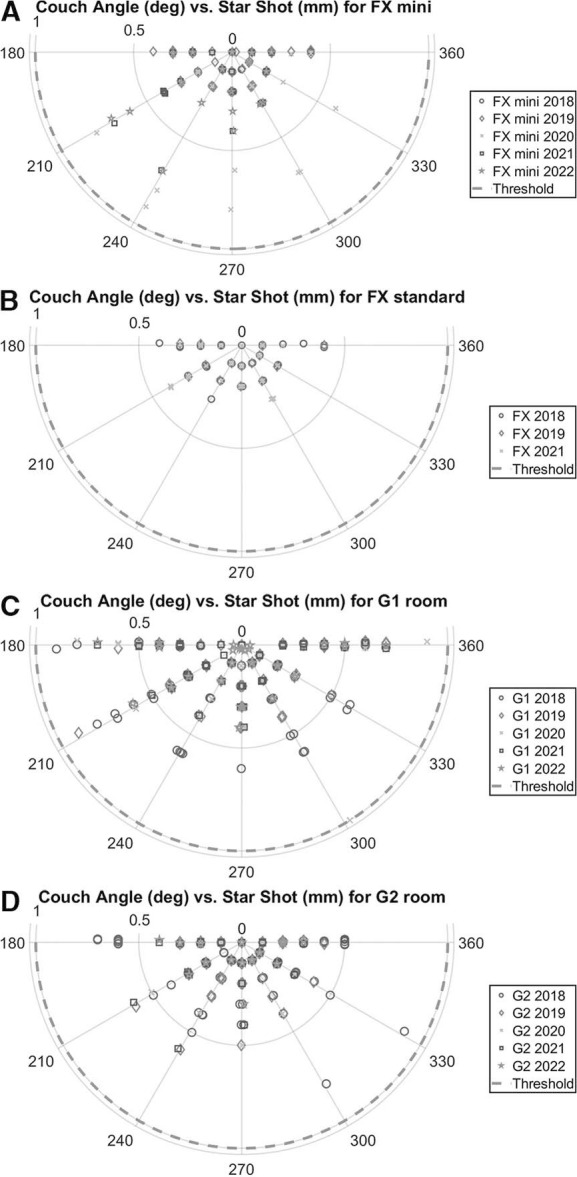
Couch star shot measurements for the FX room mini beam (A), FX room standard beam (B), G1 room (C), and G2 room (D), color-coded by year from 2018 to 2022. FX: fixed-gantry room.

The gantry and couch isocentricity or star shot measurements were analyzed in the XRV software as the closest displacement of each beam from the intersection of all beams. The radiation isocenter accuracy was 0.3 ± 0.2 mm for the gantry star shots and 0.2 ± 0.2 mm for the couch star shots for all rooms between 2018 and 2022, whereas the clinical threshold was 1.0 mm. **Figure 4** shows polar plots representing the couch angles sweeping from 180° to 360° for all rooms, including the fixed-gantry room.

**Figure 5.**
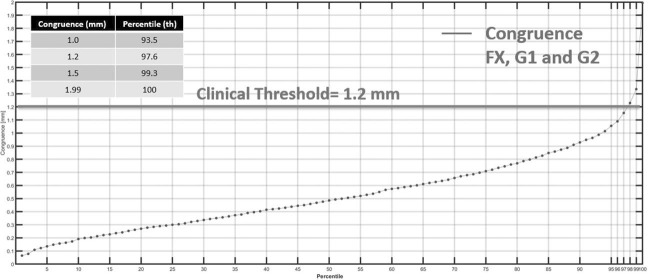
Radiation and imaging congruence for all rooms displayed according to the percentiles of all measurements. FX: fixed-gantry room.

Congruence was measured simultaneously with the gantry star shot after radiographic imaging, enabling verification of the radiation and imaging coincidence. It was 0.5 mm for the 50^th^ percentile and 1.2 mm (clinical threshold) for the 97.6^th^ percentile for the 1176 individual monthly QA measurements (including those from all 3 rooms) (**Figure 5**).

## Discussion

Monthly QA procedures for proton beam delivery systems are now established as routine. The manufacturer follows a rigorous preventative maintenance schedule in accordance with the clinical physics team to ensure that gantry and couch isocentricity results are kept less than 1.0 mm. However, when failures occurred with values exceeding the clinical threshold, treatment with the proton system was put on hold, and the components of the system, including the imaging setup, couch, and gantry, were investigated.

The American Association of Physicists in Medicine Task Group recommendations regarding machine checks are intended to be the bare minimum and apply to most centers nationwide. Centers that treat specific disease sites or patient cohorts may require the design and inclusion of more verifications or checks focused on a particular area of machine performance as part of the routine physics check.

**Figure 6.**
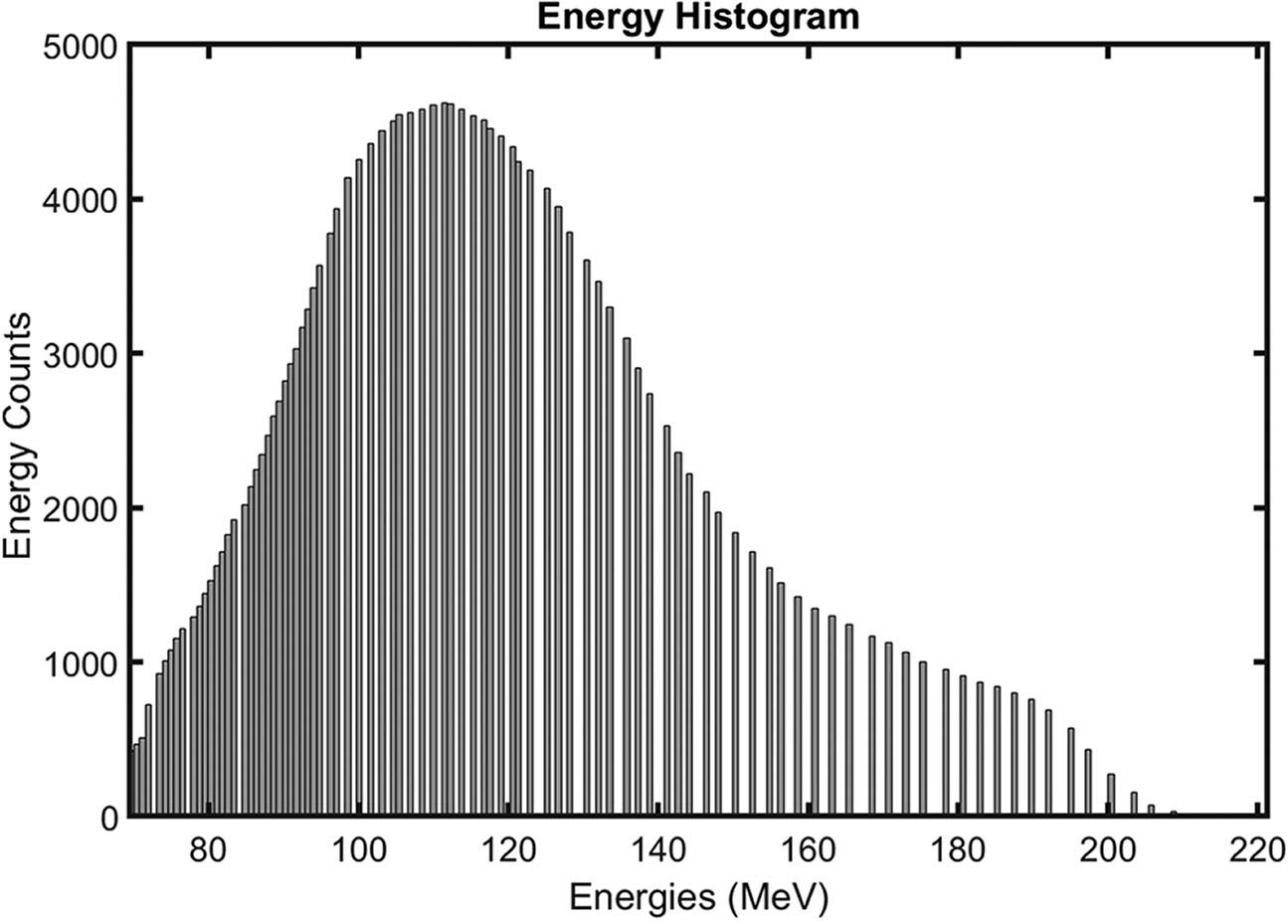
Energy histogram of the beam energy layers used in the treatment planning system between 2016 and 2021.

Our pencil-beam scanning proton therapy machine can generate proton beams within the 69.4 to 221.3 MeV range. Therefore, at its commissioning in 2015, we established a monthly QA scheme to verify 7 beam energy properties ranging from the lowest to the highest energies, including 69.4, 84.5, 100, 139.1, 180.4, 200.4, and 221.3 MeV. In a parallel study, we analyzed the beam energies used in the patients treated between 2016 and 2021, collectively totaling 217 015 energy layers from 992 patients. **Figure 6** shows that the mean energy used in the treatment planning system was 114.4 ± 26.4 MeV, corresponding to a penetration range of approximately 10 cm in water. In contrast, the highest energy of 221.3 MeV corresponds to a range of 31 cm in water. The beam energies frequently used in the treatment planning system for patient treatments should be considered for more regular verification as part of the routine machine checks to reconcile clinical needs with the routine physics checks.

## Conclusion

Augmenting an XRV-100 device with 2 in-house phantoms improved the quality of the monthly QA for our proton therapy system compared with the previous methods using radiochromic films, loose ball bearings, and foam. The digital analysis of the results also reduced the high demand for radiochromic film and the need for tedious scanning procedures. The scheme of alternating beam angles with discrete energies in the monthly QA enabled clinically meaningful verification of beam energy and gantry angle combinations while the machine performance and accuracy were being checked.
